# Supplementary material for: Semaphorin4A promotes lung cancer by activation of NF-κB pathway mediated by PlexinB1
Source: PeerJ. 2023 Oct 24;11:e16292. doi: 10.7717/peerj.16292 (PMC10607275; doi:10.7717/peerj.16292)

Figure 1B

The original blot of GAPDH in LC cells

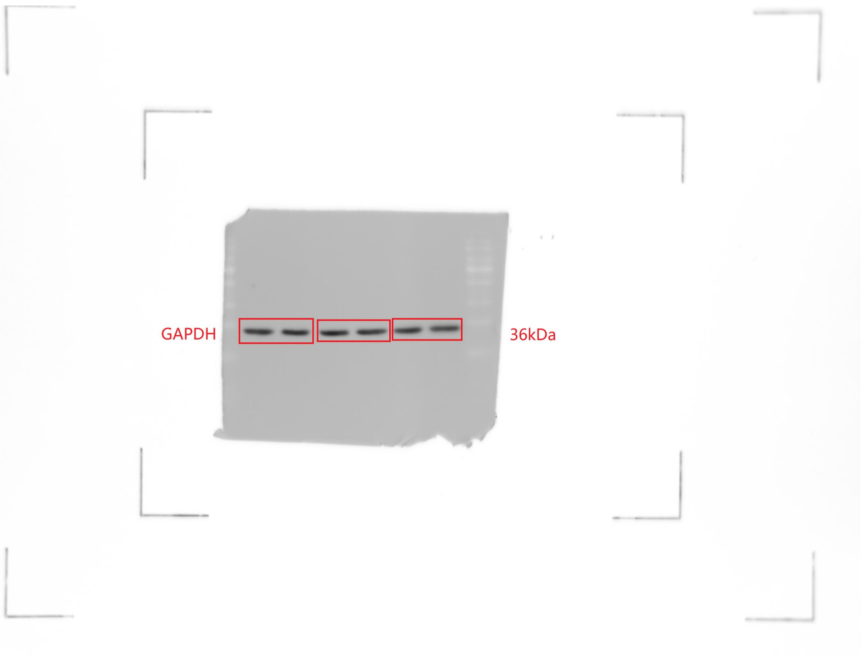

Figure 1B

The original blot of Sema4A in LC cells

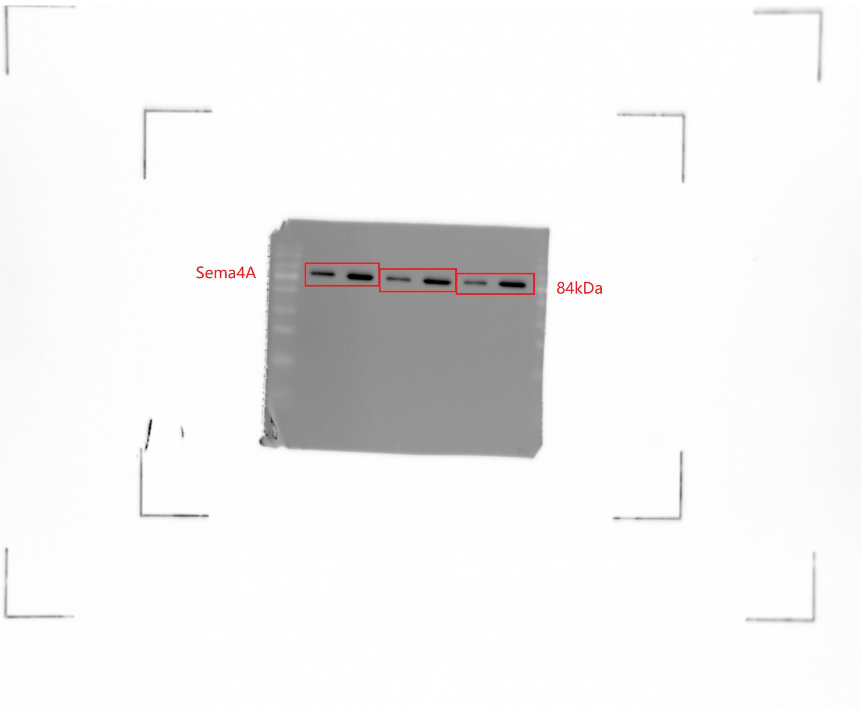

Figure 2

The original blot of GAPDH in LC cells

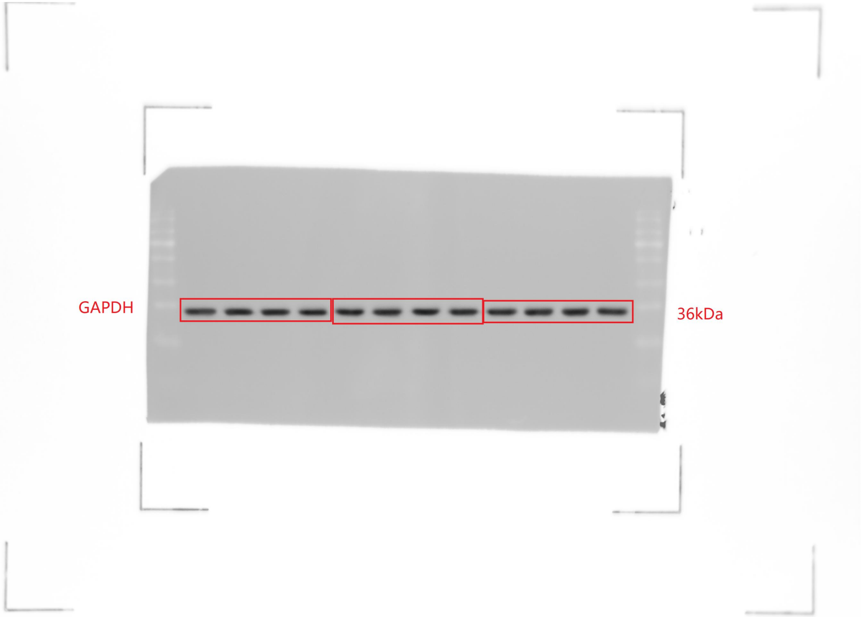

Figure 2

The original blot of NF-kB in LC cells

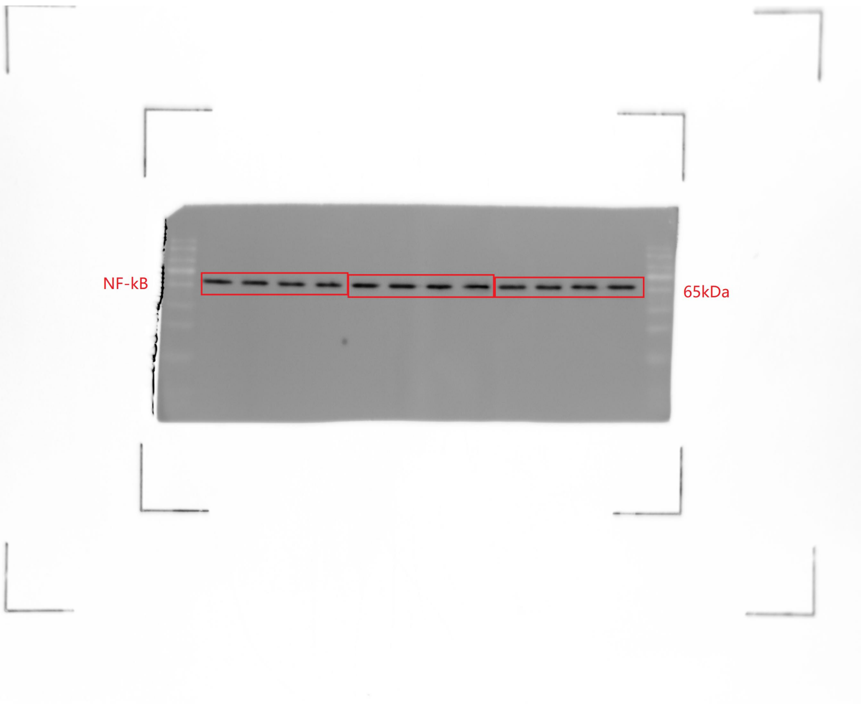

Figure 2
The original blot of p-NF-kB in LC cells

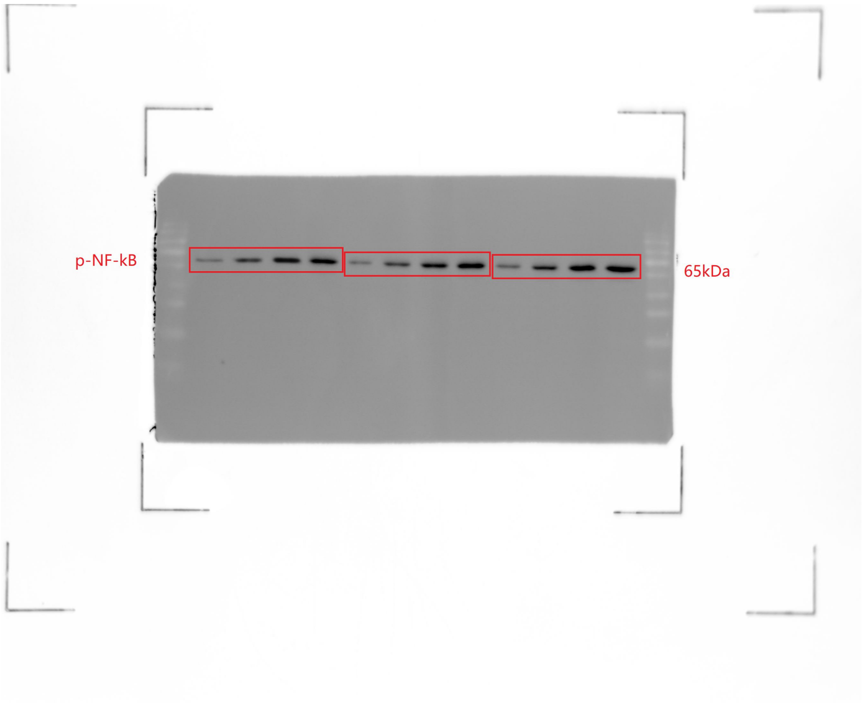

Figure 2

The original blot of Stat3 in LC cells

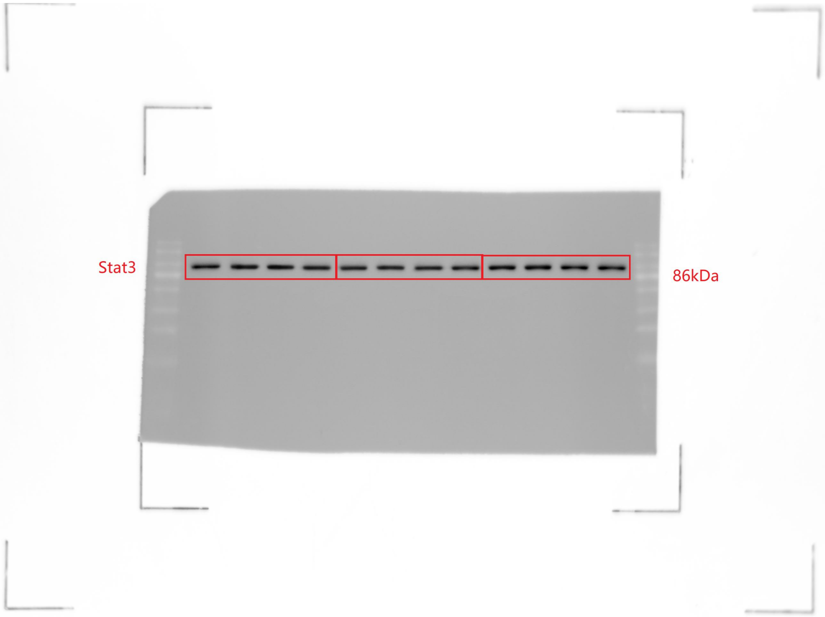

Figure 2

The original blot of p-Stat3 in LC cells

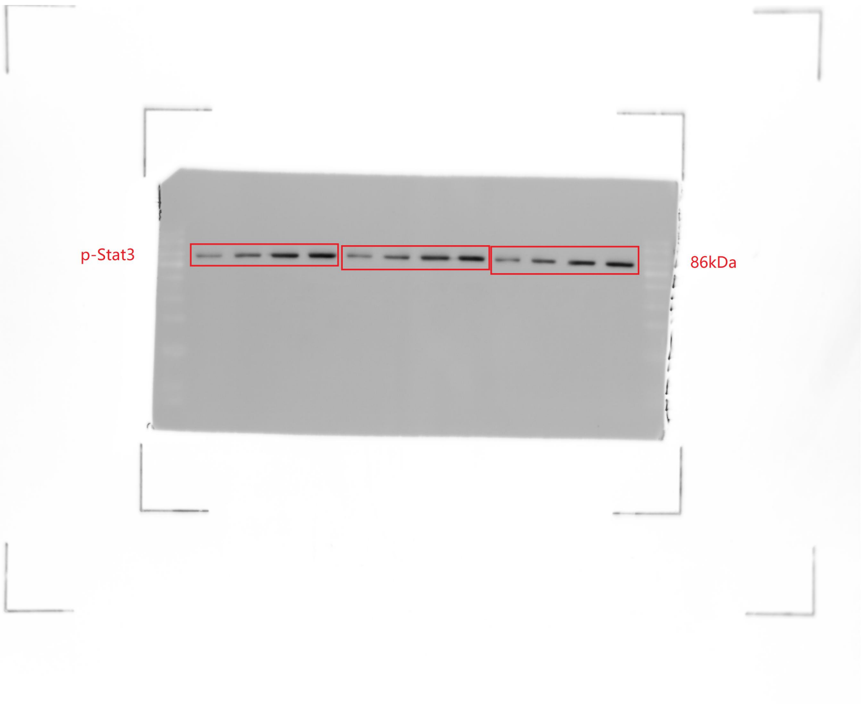

Figure 2

The original blot of MAPK in LC cells

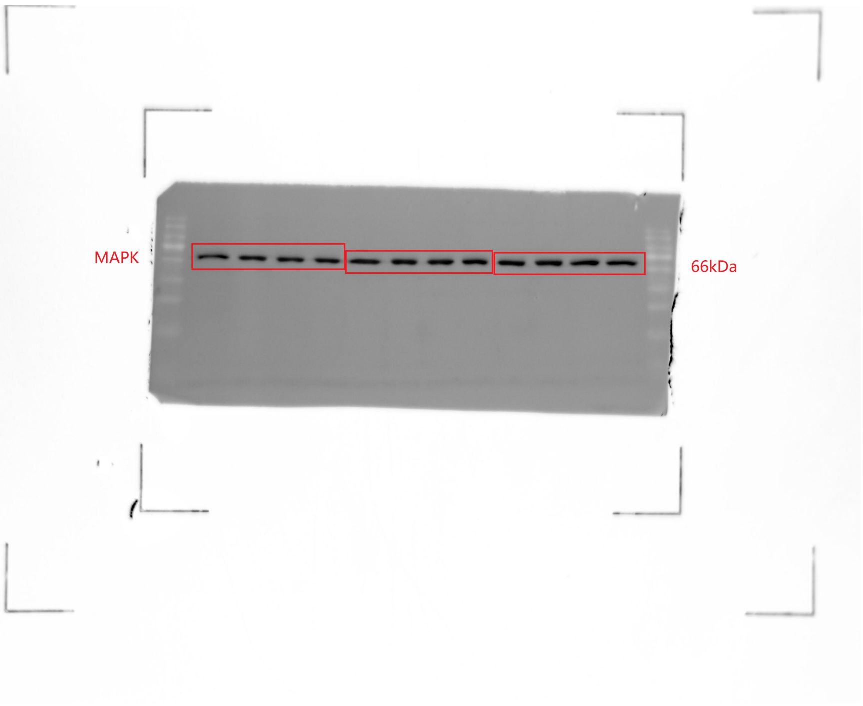

Figure 2

The original blot of p-MAPK in LC cells

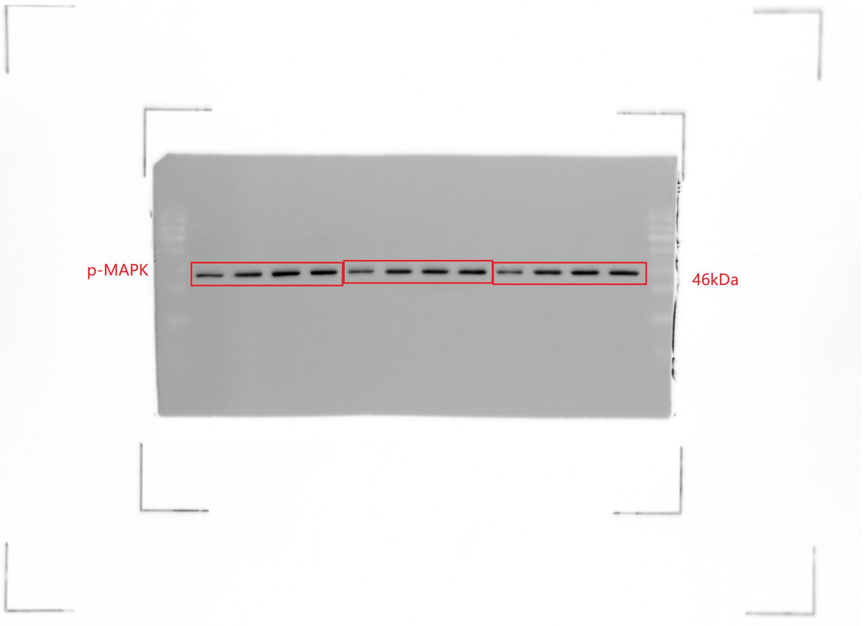

Figure 6

The original blot of GAPDH in LC cells

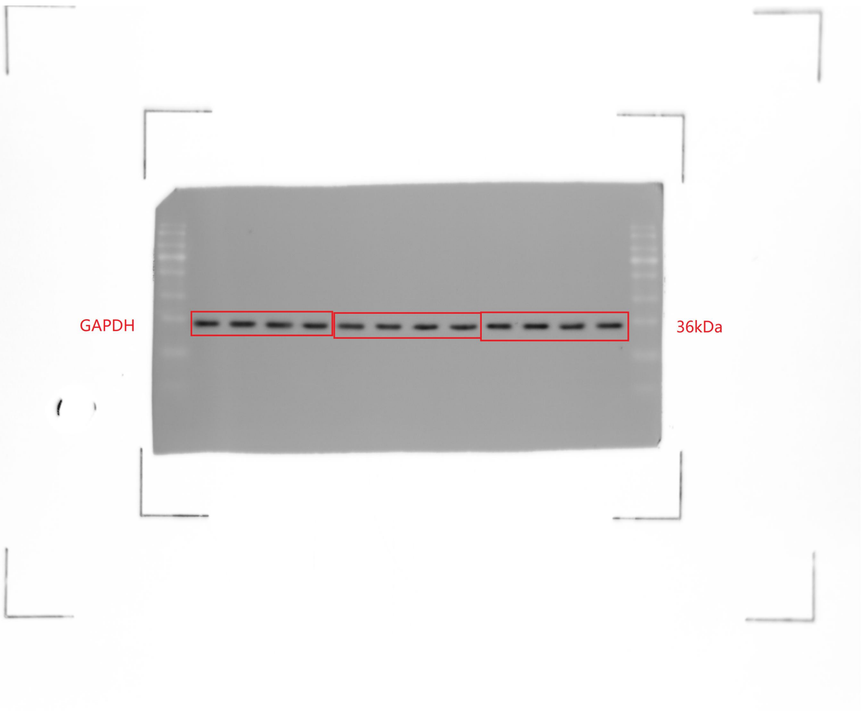

Figure 6

The original blot of NF-kB in LC cells

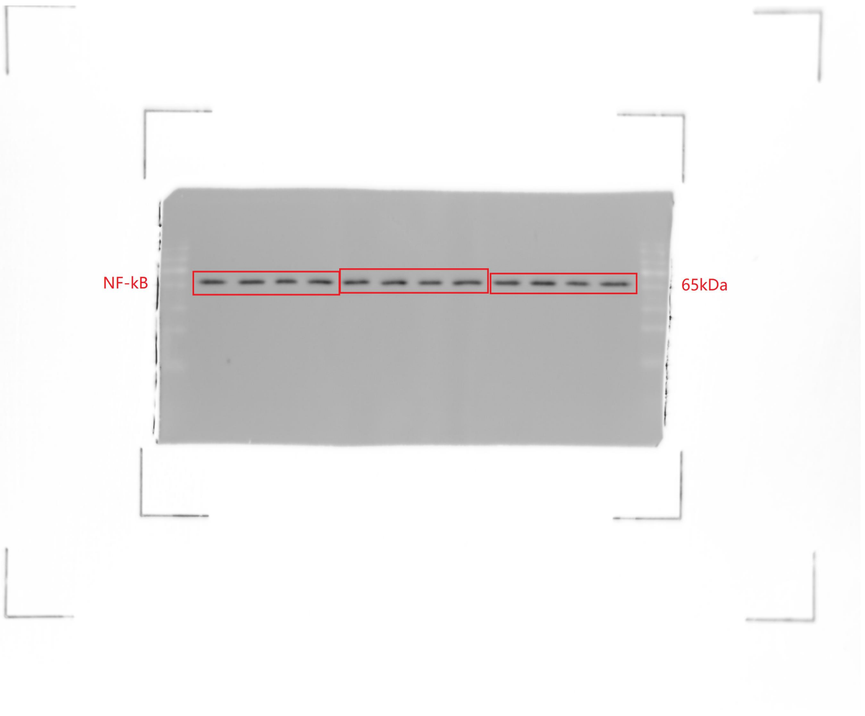

Figure 6

The original blot of p-NF-kB in LC cells

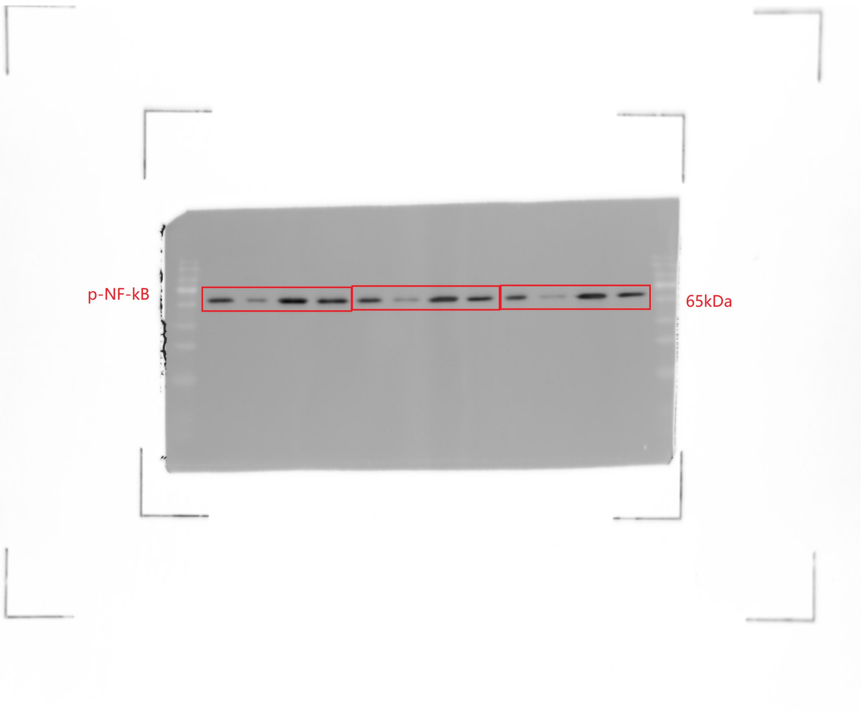

Figure 6

The original blot of Stat3 in LC cells

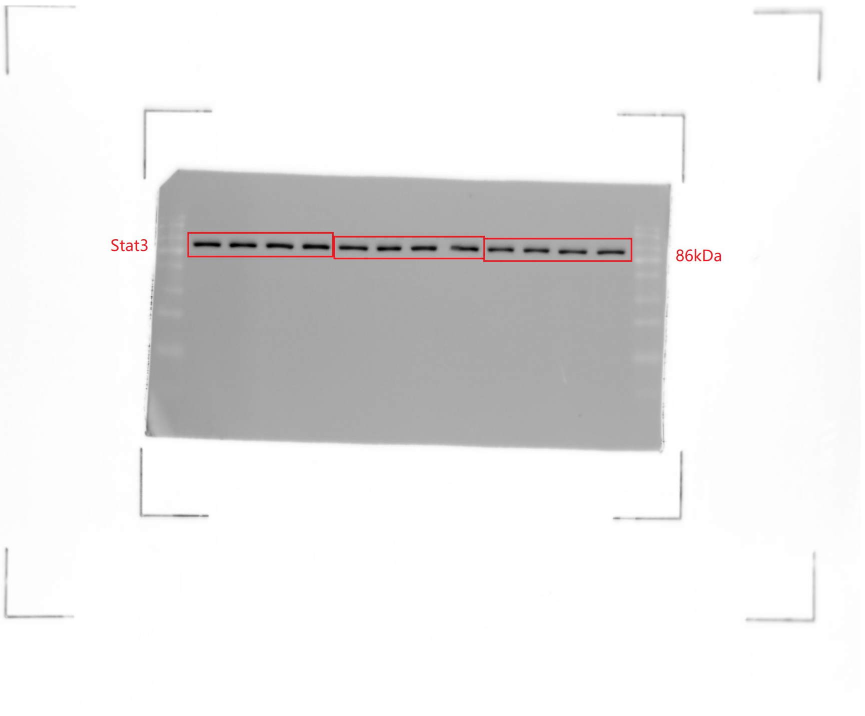

Figure 6

The original blot of p-Stat3 in LC cells

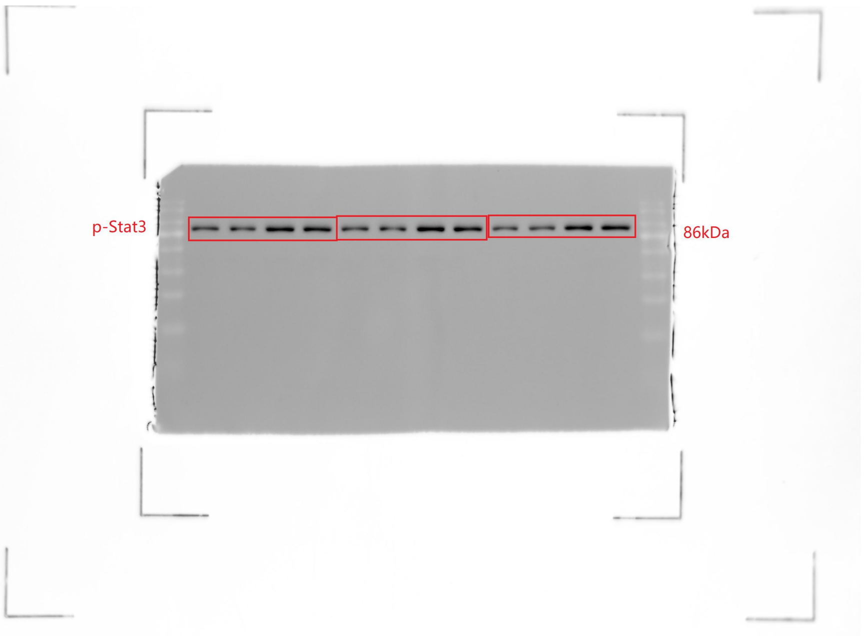

Figure 6

The original blot of MAPK in LC cells

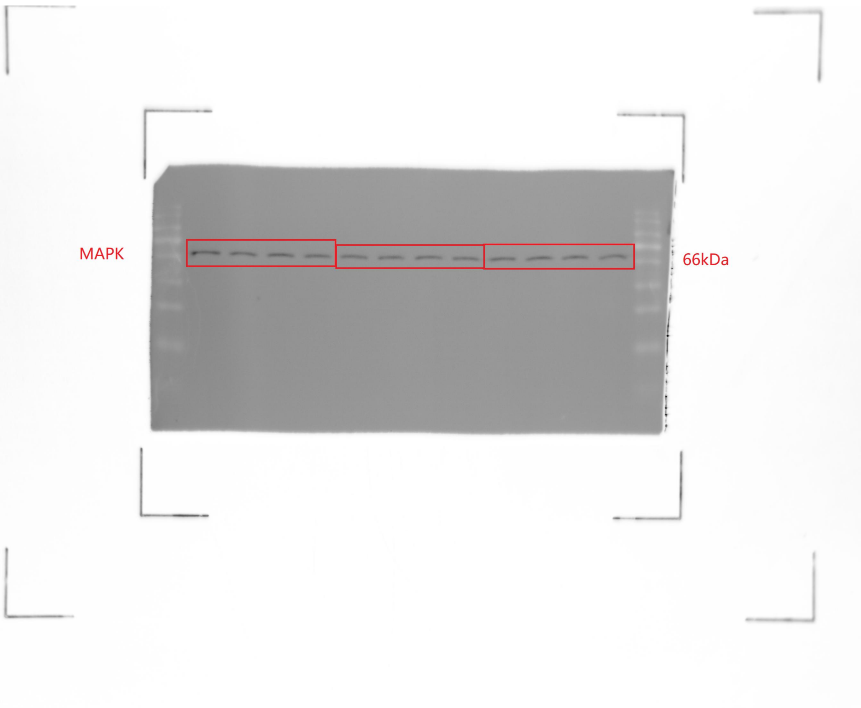

Figure 6

The original blot of p-MAPK in LC cells

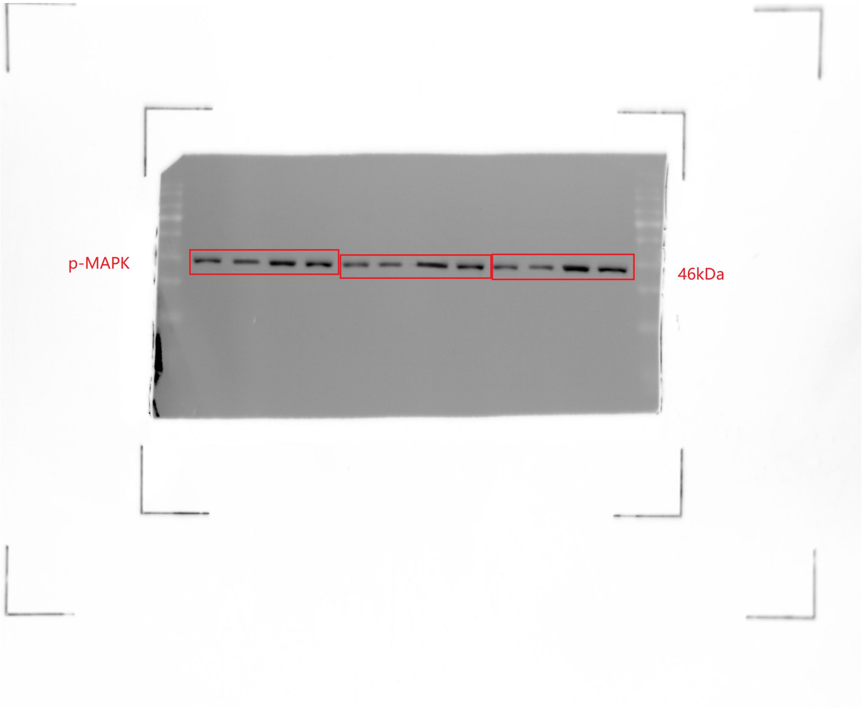

Supplement: Supplemental Information 2 [file peerj-11-16292-s002.pdf]
